# Comment to: Comparison of mechanical properties and host tissue response to OviTex™ and Strattice™ surgical meshes

**DOI:** 10.1007/s10029-023-02822-y

**Published:** 2023-06-23

**Authors:** S. J. Pacella, R. Nazerali

**Affiliations:** 1 Scripps MD Anderson Cancer Center, La Jolla, CA USA; 2 Stanford University School of Medicine, Stanford, CA USA

We congratulate Lombardi and the Allergan team [1] for their pre-clinical comparison of the reinforced biologic, Ovitex^®^ and biologic Strattice^®^, both indicated and widely used clinically for hernia repair.

The authors do appropriately indicate that “the need for adequate education and comparative studies… are especially important for informing the selection of an appropriate mesh in the clinical setting where reduction of postoperative pain, stiffness, and other complications at the surgical site is paramount for hernia patients.” We concur with the authors on this point, however, we disagree that clinical decisions should be made based on the bench testing and short-term small animal studies performed, particularly when comparative human clinical studies exist.

We would like to bring to the readers’ attention the published clinical investigations of Sivaraj et al. [2] and Goetz et al. [3], among several others [4–6]. Sivaraj et al. compared clinical outcomes following ventral hernia repair with either Ovitex (n = 36) or Strattice (n = 51) along with several other biologic products. There were no statistical differences in the cohorts with respect to patient co-morbidities, surgical characteristics or hernia type. Recurrence rates between the two cohorts were not statistically different, while overall complication rates (including recurrence) were significantly lower in the OviTex cohort compared to the Strattice cohort. Poisson Regression analysis demonstrates the overall relative risk of post-operative complications is significantly less in the OviTex cohort versus the Strattice cohort (RR = 2.64, p = 0.0182).

Goetz et al. compared OviTex to three other biologic meshes, including Strattice, in the setting of possible wound contamination. Patients with abdominal wall reconstruction, from either a ventral hernia or open abdomen closure, were included in the study. There were no differences in age, gender, BMI, operation duration, hernia size or Charlson comorbidity index in the 28 OviTex patients or 43 biologic mesh patients studied. Hernia recurrence within 24 months of repair was significantly lower in the OviTex group (3.6 vs. 28.9%; p = 0.03) while seroma requiring intervention (61.4 vs. 55.5%, p = 0.43) and operative revision (28.6 vs. 16.3%, p = 0.22) were not statistically different between groups.

We believe that the pre-clinical results presented by Lombardi et al. are likely an artifact of cutting the mesh into a non-representative 1 × 7 cm coupon sized which was placed into a subcutaneous pocket of a rat. In our experience, when appropriately placed and fixated, the OviTex layers rapidly and completely integrate into the surrounding tissue, contributing to the strength of the repair. Conversely, Strattice poorly integrates, encapsulates and never fully integrates into the surrounding host tissue. In the clinical setting, we see that the limited stretch of OviTex, compared to the relatively high stretch of Strattice, enables us to offload tension and maintain the integrity of the repair. The authors infer that “stronger is better” without accounting for the biomechanical properties of the native abdominal wall (including stretch and compliance), calling into question the clinical validity of this research.

In summary, we propose the “preferable host biologic response” is the one that delivers substantiated human clinical results. The pre-clinical work presented are incomplete and unsubstantiated. Objective and robust pre-clinical and clinical data must remain our gold standard.
